# An Analysis of the Attitude Estimation Errors Caused by the Deflections of Vertical in the Integration of Rotational INS and GNSS

**DOI:** 10.3390/s19071721

**Published:** 2019-04-10

**Authors:** Hao Xiong, Dongkai Dai, Yingwei Zhao, Xingshu Wang, Jiaxing Zheng, Dejun Zhan

**Affiliations:** College of Advanced Interdisciplinary Studies, National University of Defense Technology, Changsha 410073, China; xh_vivid@126.com (H.X.); yingweizhao@live.cn (Y.Z.); gfkdwxs@aliyun.com (X.W.); chaser89@126.com (J.Z.); zdj4444@sina.com (D.Z.)

**Keywords:** deflections of vertical, attitude estimation errors, rotational INS, INS/GNSS integration, global gravity model

## Abstract

This paper investigates the attitude estimation errors caused by the deflections of vertical (DOV) in the case of a rotational inertial navigation system (INS) integrated with a global satellite navigation system (GNSS). It has been proved theoretically and experimentally that the DOV can introduce a tilt error to the INS/GNSS integration, whereas less attention has been given to its effect to the heading estimation. In fact, due to the intercoupling characteristic of attitude errors, the heading estimation of an INS/GNSS integrated navigation system can also be affected. In this paper, first, the attitude estimation errors caused by DOV were deduced based on the INS’s error propagation functions. Then, the corresponding simulations were conducted and the results were well consistent with the theoretical analysis. Finally, a real shipborne marine test was organized with the aimed to verify the effect of DOV on attitude estimation in the rotational INS/GNSS integration, whereas the global gravity model was used for DOV compensation. The results with DOV compensation were compared with the corresponding results where the compensation was not used and showed that the heading estimation errors caused by DOV could exceed 20 arcsecs, which must be considered in high-precision application cases.

## 1. Introduction

In inertial navigation systems, accelerometers can only sense the specific forces but not the acceleration caused by the gravitational field. Thus, the gravitational information should be previously acquired to obtain the exact trajectory of the vehicles [1]. In general, a simple ellipsoid model such as WGS84, which is usually named as the normal gravity model, is employed in the solution of INS to balance the dilemma between the accuracy and the computational efficiency [2]. However, the gravity disturbance, which can be defined as the difference between actual gravity and normal gravity, is always an error source for INS. For low-precision INS such as the MEMS system, inertial sensors’ biases and random noises are the dominant error sources. Other items such as the Coriolis term, transport rate, and the lever-arm effect can be neglected, let alone the gravity disturbance [3,4]. While in high-precision ones, the random noises of the system can be relatively smaller with a significant improvement of the inertial sensors. Aside from this, the systematic errors such as fixed angle error, scale factor, inner lever-arm, and so on can be previously obtained through calibration and fine alignment [5,6]. After that, the gravity disturbances can dominate the residual errors and take on a new importance. 

The gravity disturbances can be represented by the magnitude and direction differences between the actual gravity vector and the normal one. The angular deviations of direction about the north and east axis of the local geographic frame are defined as the DOV, and the magnitude deviation is defined as the gravity anomaly, which is approximate to the vertical component of the gravity disturbance vector [7]. For the gravity anomaly, its effect is confined in the vertical channel of the INS, where the exponential errors caused by it can be easily damped by the external height information, for example, the barometer or depth gage etc. [8]. In addition, the tilt angle of vehicles is relatively small under the shipborne condition, which makes the horizontal channels decouple with the vertical channel as the errors caused by the gravity anomaly cannot transfer to the horizontal channel and affect the attitude solution [9]. Thus, when researchers investigate the effect of gravity disturbance on the INS, the DOV is of more concern, since it is the larger error source in the INS [10], whereas the gravity anomaly is generally neglected. Considering this, this paper concentrated on the influence of DOV on the INS.

For long-term inertial navigation cases, the investigation of the effect of DOV on pure inertial navigation dates back to several decades ago. In the work of Levine and Gelb, navigation errors such as velocity, position, azimuth, platform tilt, and so on were evaluated for a wide range of vehicle speeds [10]. In addition, a covariance propagation analysis method was proposed to investigate the effect of DOV theoretically. Based on the research of Levine and Gelb, some experienced stochastic models have been performed to analyze its effect on the INS as the first-order Gauss–Markov process is reluctant to reflect the nonisotropic characteristic of the DOV [11,12,13,14]. Meanwhile, different DOV compensation methods have also been proposed to further enhance the performance of the INS. A conventional way is to use gravitational gradiometers to obtain the DOV along the trajectory [15,16,17], but this method is confined in practical application due to its high cost. 

Another simple way is to use global gravity models to calculate the DOV, for example, the Earth Gravitational Model (EGM2008) [18], GGMplus [19], etc. The DOV value along the track can be obtained by interpolation from the offline database or direct computation using the spherical model [2,20,21], then the compensation can be implemented. In short-term application cases, the 3-D navigation errors caused by gravity disturbance were simulated by Jekeli, and indicated that the gravity disturbance must be taken into consideration when the decimeter level positioning accuracy is demanded over the period as short as 100s [6].

Given the complementary nature of the INS and GNSS, integrating the INS and GNSS can make the best use of each other and construct a long-term stable, self-confined, and high output frequency position–velocity–attitude navigation system [22]. In the high-precision INS/GNSS integration, the biases of inertial sensors can be estimated and corrected via the navigation Kalman filter. Thus, the DOV can dominate the residual errors and become the main error source of the system. It should be recognized that the position and velocity errors caused by the DOV can be easily damped with the aid of GNSS, however, the attitude estimation of the integrated navigation system can still be affected [5]. In the work of Grejner-Brzezinska [5,23,24], it was shown theoretically and experimentally that DOV can introduce a tilt error of vehicles, which should be compensated or modeled for a high accuracy attitude estimation. In fact, high-precision INS/GNSS integrated navigation systems have also served as vector gravimetry to measure the DOV [25,26,27,28]. In the studies of [27,28], the attitude reference decoupled from DOV was constructed with the raw gyros data of the INS for the INS/GNSS attitude error calculation. Subsequently, with the accurate modeling of DOV and inertial sensors, the DOV can be obtained by the difference between two sets of attitudes. These works show that the attitude estimation of INS/GNSS integration can be affected by the DOV, which can be separated from other error items. It should be noted that only the tilt error of platforms were of concern in those analyses, while the heading estimation error caused by the DOV was not addressed. Nevertheless, due to the intercoupling characteristic of attitude errors, the heading estimation in the INS/GNSS integration can also be affected.

This paper addressed the attitude estimation errors caused by the DOV. The remainder of the paper is organized as follows. Section 2 analyzes the effect of DOV to attitude estimation errors based on the INS’s error propagation equations. Section 3 performs the simulations, which were well consistent with the analytical results. In Section 4, a real shipborne marine test was conducted to further demonstrate the influence of DOV on the attitude estimation errors of the rotational INS/GNSS integration. Finally, our conclusions are drawn in Section 5.

## 2. Theoretical Analysis

### 2.1. The Definition of the Coordinates System and DOV

**Figure 1:**
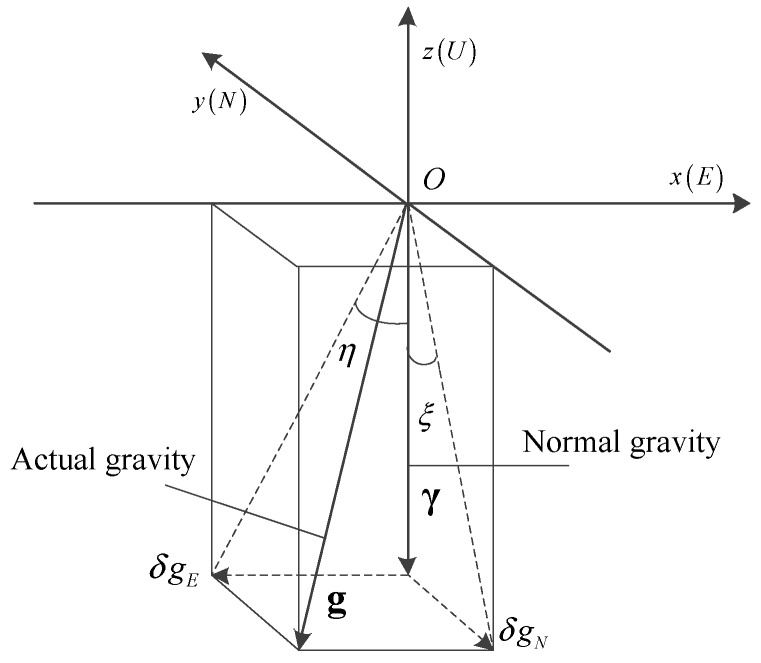
The definition of the coordinates system and DOV.

To avoid confusion, the definition of DOV and the coordinate systems used in this paper are first introduced. As shown in Figure 1, the local geodetic coordinate is defined as the navigation frame (*n*-frame), where the origin is at the measurement site and the *z*-axis points toward the exterior of the ellipsoid normal (U). The *x*-axis points toward the east (E) and the *y*-axis points toward the north (N), which complete an orthogonal, right-hand rectangular coordinate system (ENU frame). 

The gravity disturbance δg is defined as the difference between actual gravity g and the normal gravity γ, which can be expressed as.

(1) $$\delta g=g-\gamma ={\left[\begin{array}{ccc} \delta g_{E} & \delta g_{N} & \Delta g \end{array}\right]}^{T}$$

where the east and north components of gravity disturbance are denoted by $\delta g_{E}$ and $\delta g_{N}$, and Δg is the vertical gravity disturbance or gravity anomaly. The relationship between the horizontal gravity disturbance and the DOV are shown as follows [7]

(2) $$\xi \approx \tan\xi =-\frac{\delta g_{N}}{g};\;\eta \approx \tan\eta =-\frac{\delta g_{E}}{g};$$

where g is the magnitude of the normal gravity and can be calculated by using the WGS84 model directly. The north–south and east–west angular components of the DOV are denoted by ξ and η, respectively. Substituting Equation (2) into Equation (1), the gravity disturbance can be rewritten as

(3) $$\delta g=\left[\begin{array}{ccc} -\eta g & -\xi g & \Delta g \end{array}\right]$$

### 2.2. The Attitude Estimation Errors Caused by DOV in the Integration of GNSS and Rotational INS

The state vector of the conventional INS/GNSS integrated navigation system is [22,29]

(4) $$\begin{array}{c} \delta x=[\begin{array}{ccccccc} \phi_{E} & \phi_{N} & \phi_{U} & \delta v_{E} & \delta v_{N} & \delta v_{U} & \delta \lambda \end{array}\\ {\begin{array}{cccccccc} \delta L & \delta h & \varepsilon_{x}^{b} & \varepsilon_{y}^{b} & \varepsilon_{z}^{b} & \nabla_{x}^{b} & \nabla_{y}^{b} & \nabla_{z}^{b} \end{array}]}_{15\times 1}^{T} \end{array}$$

where $\phi_{E}$, $\phi_{N}$, $\phi_{U}$ are the east, north, and heading components of the attitude errors expressed as $\Phi^{n}$, respectively; $\delta v_{E}$, $\delta v_{N}$, $\delta v_{U}$ indicate the east, north, vertical velocity errors expressed as $\delta V^{n}$, respectively; δλ, δL, δh are the position errors expressed in longitude, latitude, and height, respectively; $\varepsilon_{x}^{b}$, $\varepsilon_{y}^{b}$, $\varepsilon_{z}^{b}$ represent the gyro biases in the body frame (*b*-frame), respectively; and $\nabla_{x}^{b}$, $\nabla_{y}^{b}$, $\nabla_{z}^{b}$ are the accelerometer biases in the *b*-frame.

The velocity error transition function of INS in the *n*-frame is given as

(5) $$\delta {\dot{V}}^{n}=f^{n}\times \Phi^{n}-\left(2\delta \omega_{ie}^{n}+\delta \omega_{en}^{n}\right)\times V^{n}-\left(2\omega_{ie}^{n}+\omega_{en}^{n}\right)\times \delta V^{n}+C_{b}^{n}\nabla^{b}+\delta g$$

where $V^{n}$ and $f^{n}$ are the velocity of INS and specific forces, respectively; $\omega_{ie}^{n}$ is the earth rotation rate with respect to the inertial frame (*i*-frame) expressed in the *n*-frame; $\omega_{en}^{n}$ represents the rotation rate of the *n*-frame with respect to the Earth-centric fixed frame (*e*-frame); $\delta \omega_{ie}^{n}$ and $\delta \omega_{en}^{n}$ are the relevant errors of $\omega_{ie}^{n}$ and $\omega_{en}^{n}$; and $C_{b}^{n}$ is the direction cosine matrix (DCM) from the *b*-frame to *n*-frame. 

In INS/GNSS integration, the GNSS receiver can output accurate velocity and position information directly, thus variables such as $V^{n}$, $\omega_{ie}^{n}$, and $\omega_{en}^{n}$ can be considered as the known parameters. In addition, the position and velocity errors of INS can be obtained when adopting the GNSS updates as the reference values, then the $\delta {\dot{V}}^{n}$, $\delta V^{n}$, $\delta \omega_{ie}^{n}$, and $\omega_{en}^{n}$ related to them can also be calculated and treated as the known parameters. Rearranging Equation (5), we can obtain

(6) $$y=f^{n}\times \Phi^{n}+\nabla^{n}+\delta g$$

where $y=\delta {\dot{V}}^{n}+\left(2\delta \omega_{ie}^{n}+\delta \omega_{en}^{n}\right)\times V^{n}+\left(2\omega_{ie}^{n}+\omega_{en}^{n}\right)\times \delta V^{n}$ is the linear combination of known parameters. Expanding Equation (6), the east and north components can be given as

(7) $$y_{E}=\phi_{U}f_{N}-f_{U}\phi_{N}+\nabla_{E}-\delta g_{E}$$

(8) $$y_{N}=\phi_{E}f_{U}-f_{E}\phi_{U}+\nabla_{N}-\delta g_{N}$$

where $y_{E}$ and $y_{N}$ are the east and north components of **y**, respectively; $\nabla_{E}$ and $\nabla_{N}$ are the east and north components of equivalent accelerometer biases in the *n*-frame, respectively; $f_{E}$, $f_{N}$, and $f_{U}$ represent the east, north, and vertical components of $f^{n}$, respectively. Since the vertical channel is decoupled from the horizontal channels, only the east and north components were analyzed in this paper.

Assume that the vehicle is static or cruising at an approximately constant velocity that can be easily satisfied in the shipborne applications. Then, there exist $f_{E}\approx 0$, $f_{N}\approx 0$, and $f_{U}\approx g$; substituting this into Equation (7) and (8), the east and north components of the attitude errors can be expressed as

(9) $$\phi_{E}=\frac{y_{N}-\nabla_{N}-\delta g_{N}}{g}$$

(10) $$\phi_{N}=\frac{\nabla_{E}-y_{E}+\delta g_{E}}{g}$$

For the high-precision INS with the single-axis rotation modulation, the biases of inertial sensors in the horizontal-axis can be compensated by the periodical rotation [29]. Furthermore, in the INS/GNSS integration case, the observability of the system can also be improved significantly for the rotation modulation, then $\nabla_{E}$, $\nabla_{N}$ can be estimated and treated as the known parameters. Combined with Equation (2), Equations (9) and (10) can be rewritten as

(11) $$\phi_{E}={\hat{\phi }}_{E}+\delta \phi_{E}=\frac{y_{N}-\nabla_{N}}{g}+\xi$$

(12) $$\phi_{N}={\hat{\phi }}_{N}+\delta \phi_{N}=\frac{\nabla_{E}-y_{E}}{g}-\eta$$

where $\phi_{E}$ can be divided into two parts. Part ${\hat{\phi }}_{E}$ is independent of DOV and is composed of the already-known parameters, which can be estimated and corrected via Kalman filtering. The residual $\delta \phi_{E}$ denotes the east component of the attitude estimation errors caused by DOV, which is equal to ξ. The same for $\phi_{N}$, it can be divided into ${\hat{\phi }}_{N}$ and $\delta \phi_{N}$, where $\delta \phi_{N}$ is the north component of the attitude estimation errors caused by DOV, and is negatively related to η.

The attitude error transition function of INS in the *n*-frame can be expressed as

(13) $$\dot{\Phi }=\Phi \times \left(\omega_{ie}^{n}+\omega_{en}^{n}\right)+\delta \omega_{ie}^{n}+\delta \omega_{en}^{n}-\varepsilon^{n}-w^{n}$$

where $\dot{\Phi }$ is the time differential term of Φ; $\varepsilon^{n}$ is the equivalent biases of gyros in the *n*-frame; and $w^{n}$ is the random noise of equivalent gyros in the *n*-frame. Since $\delta \omega_{ie}^{n}$ and $\delta \omega_{en}^{n}$ are the known parameters in the INS/GNSS integration and are relatively small compared to other error terms, it was reasonable to neglect their effect in the following analysis. Expanding Equation (13), the corresponding east, north, and vertical components can be shown as follows:

(14) $${\dot{\phi }}_{E}=\left(\Omega_{U}+\omega_{U}\right)\phi_{N}-\left(\Omega_{N}+\omega_{N}\right)\phi_{U}-\varepsilon_{E}-w_{E}$$

(15) $${\dot{\phi }}_{N}=-\left(\Omega_{U}+\omega_{U}\right)\phi_{E}+\omega_{E}\phi_{U}-\varepsilon_{N}-w_{N}$$

(16) $${\dot{\phi }}_{U}=\left(\Omega_{N}+\omega_{N}\right)\phi_{E}-\omega_{E}\phi_{N}-\varepsilon_{U}-w_{U}$$

where $\varepsilon_{E}$, $\varepsilon_{N}$, and $\varepsilon_{U}$ indicate the east, north, and vertical components of $\varepsilon^{n}$, respectively; $\Omega_{N}$ and $\Omega_{U}$ are the north and vertical components of $\omega_{ie}^{n}$ ($\Omega_{E}=0$); and $\omega_{E}$, $\omega_{N}$, and $\omega_{U}$ represent the east, north, and vertical components of $\omega_{en}^{n}$. $w_{E}$, $w_{N}$, $w_{U}$ indicate the east, north, and vertical components of $w^{n}$, respectively, which satisfy that

(17) $$\sigma_{i}=E\left[w_{i}w_{i}^{T}\right]\left(i=E,N,U\right)$$

where $\sigma_{i}\left(i=E,N,U\right)$ is the variance of the random noises of the east, north, and vertical gyro. 

From Equations (14)–(16), it can be clearly seen that $\phi_{E}$, $\phi_{N}$, $\phi_{U}$ are coupled with each other. Thus, when the DOV introduces the tilt error of platforms, the heading estimation can also be affected. Rearranging Equations (14) and (15), we can obtain

(18) $$\phi_{U}=Z_{1}-\frac{w_{E}}{\Omega_{N}+\omega_{N}}$$

(19) $$\phi_{U}=Z_{2}+\frac{w_{N}}{\omega_{E}}$$

where $Z_{1}=\frac{\left(\Omega_{U}+\omega_{U}\right)\phi_{N}-{\dot{\phi }}_{E}-\varepsilon_{E}}{\Omega_{N}+\omega_{N}}$, $Z_{2}=\frac{{\dot{\phi }}_{N}+\left(\Omega_{U}+\omega_{U}\right)\phi_{E}+\varepsilon_{N}}{\omega_{E}}$.

It can be seen that the heading error can be calculated from both Equations (18) and (19). As is widely known, a Kalman filter is an unbiased, minimum variance, and linear stochastic process [30]. Thus, when deriving the heading error of the INS, both the information of Equations (18) and (19) should be considered to approximate the optimal results of the navigation Kalman filter. Combined with Equation (17), the estimation of the minimum variance of the heading error can be obtained

(20) $$\phi_{U}=\frac{{\left(\Omega_{N}+\omega_{N}\right)}^{2}\sigma_{N}}{{\left(\Omega_{N}+\omega_{N}\right)}^{2}\sigma_{N}+\omega_{E}^{2}\sigma_{E}}Z_{1}+\frac{\omega_{E}^{2}\sigma_{E}}{{\left(\Omega_{N}+\omega_{N}\right)}^{2}\sigma_{N}+\omega_{E}^{2}\sigma_{E}}Z_{2}$$

To be simplified, assuming that $\sigma_{E}=\sigma_{N}$, then Equation (20) can be written as

(21) $$\phi_{U}=\frac{{\left(\Omega_{N}+\omega_{N}\right)}^{2}}{{\left(\Omega_{N}+\omega_{N}\right)}^{2}+\omega_{E}^{2}}Z_{1}+\frac{\omega_{E}^{2}}{{\left(\Omega_{N}+\omega_{N}\right)}^{2}+\omega_{E}^{2}}Z_{2}$$

It should be noted that the speed of the vehicles is limited in shipborne application cases, which makes the magnitude of $\omega_{en}^{n}$ much smaller than that of $\omega_{ie}^{n}$. For example, assuming that a ship cruises toward the north with a velocity of 20 knots/h where the latitude is 45° N. Then, it can be calculated that the magnitude of $\omega_{E}$ and $\Omega_{N}$ are 0.33 °/h and 10.64 °/h, respectively, which satisfies

(22) $$\left(\Omega_{N}+\omega_{N}\right)>>\omega_{E}$$

thus, Equation (21) can be approximated as

(23) $$\phi_{U}\approx \frac{{\left(\Omega_{N}+\omega_{N}\right)}^{2}}{{\left(\Omega_{N}+\omega_{N}\right)}^{2}+\omega_{E}^{2}}Z_{1}\approx Z_{1}$$

then, the heading estimation error $\delta \phi_{U}$ caused by the DOV can be derived through the perturbation of Equation (23), where

(24) $$\delta \phi_{U}\approx \delta Z_{1}=\frac{\left(\Omega_{U}+\omega_{U}\right)\delta \phi_{N}-\delta {\dot{\phi }}_{E}-\delta \varepsilon_{E}}{\Omega_{N}+\omega_{N}}$$

In the rotational INS/GNSS integrated navigation system, the biases of inertial sensors in the horizontal axis can be estimated precisely and treated as known parameters, thus there exists

(25) $$\delta \varepsilon_{E}=0$$

combined with Equations (11), (12) and (25), Equation (24) can be rewritten as

(26) $$\delta \phi_{U}=-\frac{\left(\Omega_{U}+\omega_{U}\right)\eta }{\Omega_{N}+\omega_{N}}-\frac{\dot{\xi }}{\Omega_{N}+\omega_{N}}$$

As above-mentioned, the speed of the vehicles (ship) is limited, where $\omega_{N}$ ($\omega_{U}$) is much smaller than $\Omega_{N}$ ($\Omega_{U}$) and can be neglected, then Equation (26) can be further approximated as

(27) $$\delta \phi_{U}\approx \delta \phi_{U}^{1}+\delta \phi_{U}^{2}=-\eta \tan L-\frac{\dot{\xi }\sec L}{\omega_{ie}}$$

where $\omega_{ie}$ is the magnitude of $\omega_{ie}^{n}$. $\delta \phi_{U}^{1}$ and $\delta \phi_{U}^{2}$ are the heading estimation errors caused by ξ and η, respectively, which can be expressed as

(28) $$\delta \phi_{U}^{1}=-\eta \tan L$$

(29) $$\delta \phi_{U}^{2}=-\frac{\dot{\xi }\sec L}{\omega_{ie}}$$

From Equations (28) and (29), it can be seen clearly that $\delta \phi_{U}$ is negatively correlated with η and the time difference of ξ along the track.

## 3. Simulation

In this section, some simulations were conducted to investigate the effect of DOV to attitude estimation in the high-precision INS/GNSS integration. These simulations were carried out with initialization as follows:

(1) The sampling frequency of the INS and GNSS were 20 Hz and 1 Hz, respectively, and the period of the iteration of the Kalman filter was 1 s, which is consistent with the GNSS output frequency;

(2) The initial position was (22° N, 113° E, 0 m);

(3) The vehicle (survey ship) was static at the beginning and subsequently accelerated to 10 m/s in the northeast direction within 2 minutes. After that, the survey ship will cruise at this constant speed. The total simulation time was set to 40 h.

The specifications of the high-precision INS and GNSS are shown in Table 1.

The high-precision INS simulated in this section represented the INS composed of the high-quality ring laser gyros (RLGs) and quartz accelerometers (QAs). Furthermore, the single-axis rotation modulation was implemented to further improve the navigation accuracy. The rotation speed of the INS was set to 18 °/s and the static time was 300 s, which is consistent with the following experiments. Since the inertial measurement unit (IMU) was well calibrated, other error items such as lever-arm effect, fixed angle error, scale factor error could be neglected, and only the biases and random noises of inertial sensors were considered in the simulation. For GNSS, the position and velocity accuracy in the horizontal channel was set to 2 m and 0.03 m/s, respectively, which is typical of single point positioning accuracy in dual-frequency pseudorange positioning mode [31]. Due to the worse dilution of precision (DOP) in the vertical direction, the position and velocity errors in the vertical axis were two times larger than that of the horizontal axis. 

In the simulation, two sets of inertial data were generated for processing. One set was generated by using normal gravity, whereas in another set, the DOV was considered. Then, the effect of DOV was evaluated by the making difference between these two attitude results of INS/GNSS integration. Subsequently, to reduce the effect of the randomness of white noise to the attitude estimation, these two simulations used an identical white noise sequence to generate the simulated INS and GNSS data.

### 3.1. The Attitude Estimation Errors Caused by η

In this section, the attitude estimation errors caused by η and ξ were investigated respectively. For simplification, the DOV was set to vary in a sinusoidal form along the track. The theoretical and simulation attitude estimation errors were compared for analysis, where the theoretical results were calculated by Equations (11) and (12) and Equations (28) and (29), while the simulation results represent the value obtained via the navigation Kalman filter. 

**Figure 2:**
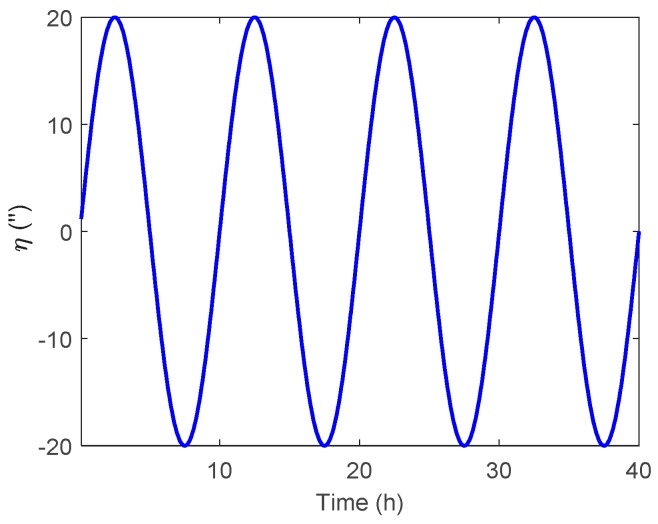
The simulated η along the track.

**Figure 3:**
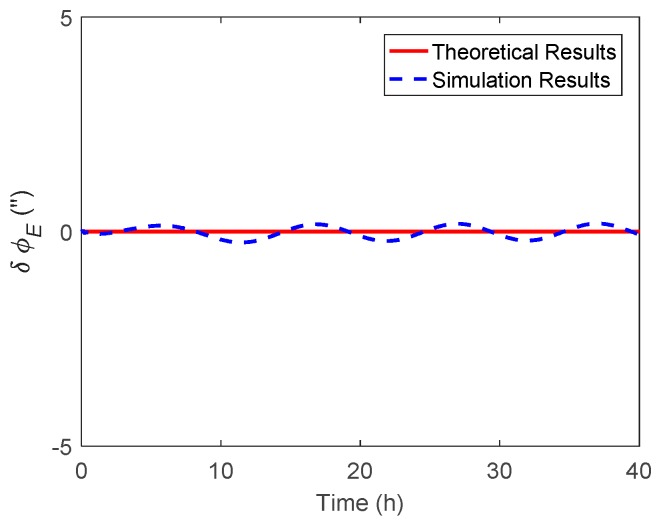
The theoretical and simulation results of $\delta \phi_{E}$ caused by η.

**Figure 4:**
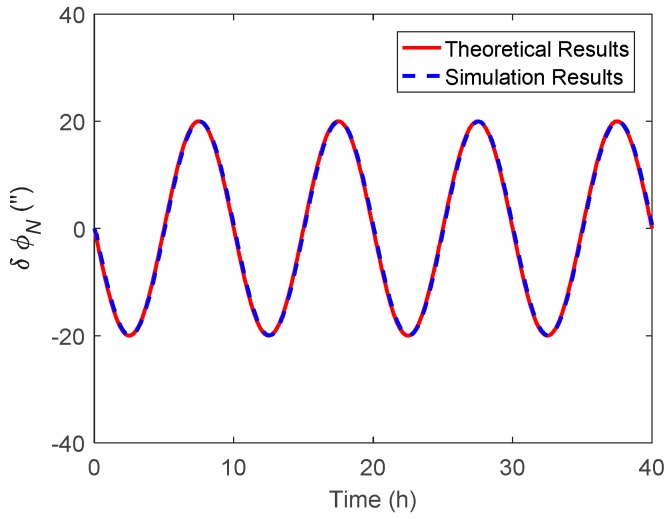
The theoretical and simulation results of $\delta \phi_{N}$ caused by η.

**Figure 5:**
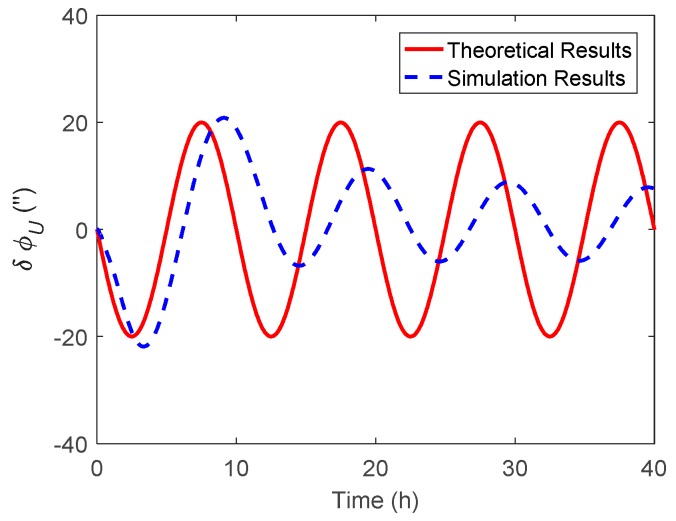
The theoretical and simulation results of $\delta \phi_{U}$ caused by η.

The simulated η along the track is shown in Figure 2, where the amplitude and period of the signal were 20 arcsecs and 10 h. The corresponding theoretical and simulation results of $\delta \phi_{E}$, $\delta \phi_{N}$ and $\delta \phi_{U}$ caused by η are shown in Figure 3, Figure 4 and Figure 5 respectively.

As shown in Figure 3 and Figure 4, the $\delta \phi_{E}$ caused by η was relatively small and less than 0.2′, whereas the $\delta \phi_{N}$ behaved as a sinusoidal form and the amplitude could reach 20 arcsecs. The simulation results of $\delta \phi_{E}$ and $\delta \phi_{N}$ were well consistent with that of the theoretical results. The heading estimation error $\delta \phi_{U}$ caused by η is shown in Figure 5, where the simulation error was also shown as a sinusoidal form, and the amplitude could reach 10 arcsec and was attenuated with time. It should be noted that there was a slight difference between the theoretical and simulation results, especially for the heading estimation, which may have several explanations. First, the theoretical results were derived upon the INS’s error propagation functions, so some error items were neglected for simplification, which may have led to the inaccuracy of the theoretical results. Second, as shown in the theoretical analysis, the DOV will first introduce the tilt error, which is then coupled into the heading error through the attitude error transition in Equation (13). Unlike the horizontal attitudes, the heading estimation is affected by the DOV indirectly. Furthermore, as is well recognized, the observability of the heading is much weaker than that of the horizontal attitudes, so the navigation Kalman filter may need more time to track the DOV signal in the heading direction. Thus, the response delay is relatively larger for the heading estimation. 

### 3.2. The Attitude Estimation Errors Caused by ξ

**Figure 6:**
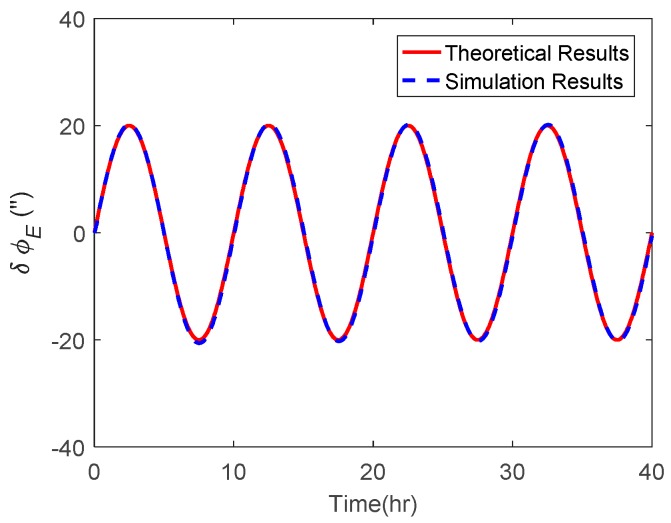
The theoretical and simulation results of $\delta \phi_{E}$ caused by ξ.

**Figure 7:**
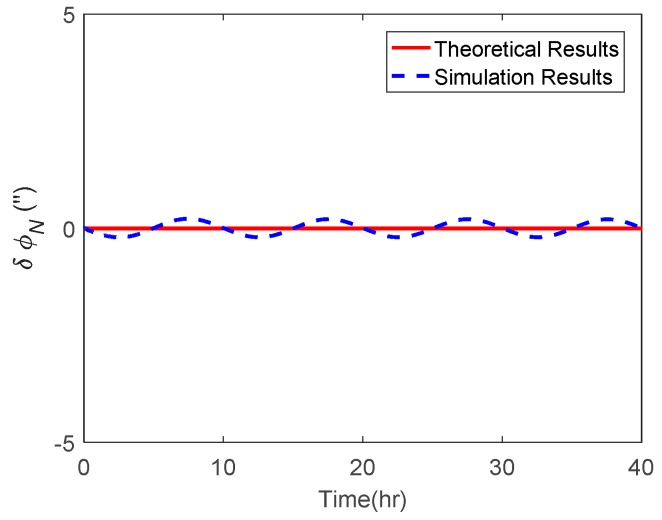
The theoretical and simulation results of $\delta \phi_{N}$ caused by ξ.

**Figure 8:**
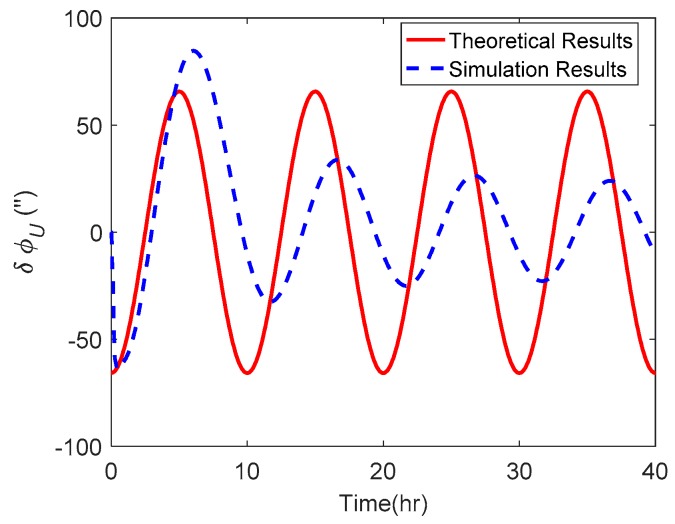
The theoretical and simulation results of $\delta \phi_{U}$ caused by ξ.

The attitude estimation errors caused by ξ were also investigated. As above, the ξ along the track was also set to vary in a sinusoidal form along the track. The corresponding theoretical and simulation results of $\delta \phi_{E}$, $\delta \phi_{N}$ and $\delta \phi_{U}$ caused by ξ are shown in Figure 6, Figure 7 and Figure 8 respectively.

As shown in Figure 6 and Figure 7, the $\delta \phi_{E}$ caused by ξ behaved as a sinusoidal form and the amplitude could reach 20 arcsecs, whereas the $\delta \phi_{N}$ was relatively small and less than 0.2′. The simulation results of $\delta \phi_{E}$ and $\delta \phi_{N}$ were well consistent with the theoretical results. From Figure 8, it can be seen that the simulation results of $\delta \phi_{U}$ caused by ξ also behaved as a sinusoidal form, where the amplitude could exceed 60 arcsecs and was attenuated with time. Due to the same reason stated in the above subsection, the simulation results of $\delta \phi_{U}$ caused by ξ showed a slight difference to the theoretical results. 

## 4. Shipborne Marine Test

### 4.1. Data Description

**Figure 9:**
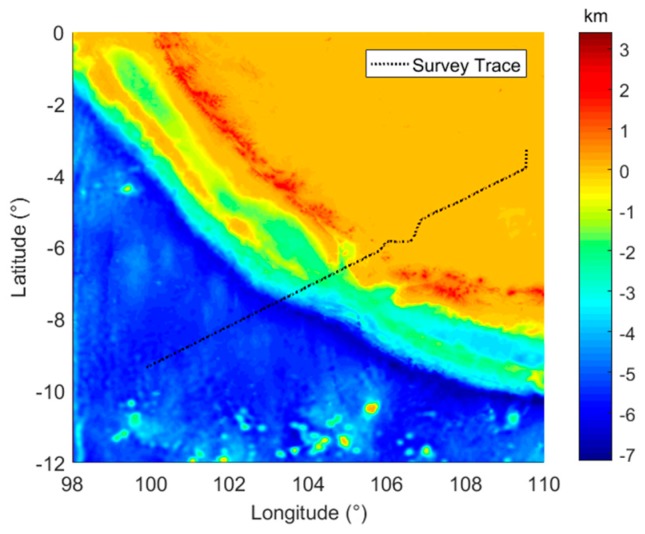
The survey line and topography of the survey region.

The ship marine test was conducted around the Strait of Malacca in October 2018. As shown in Figure 9, the survey line was about 1200 km and approximately toward the northeast. The total survey time lasted for 40 hours. Since there is a continental slope and several islands located in the survey region, the topography in the survey region changes dramatically, which can fluctuate between −7–3 km. 

**Figure 10:**
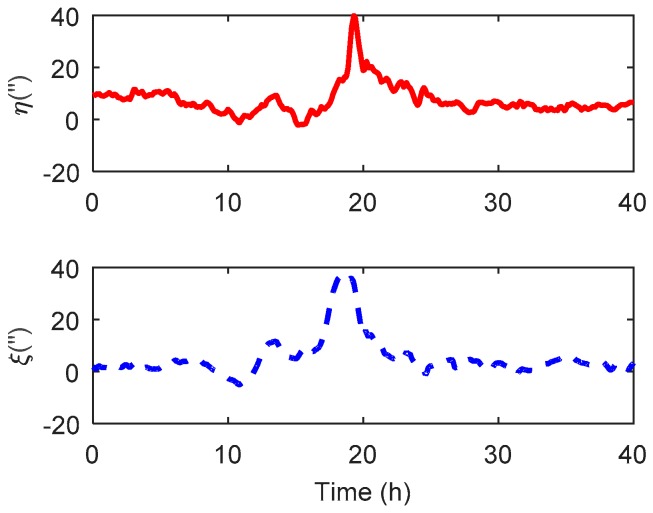
The DOV distribution obtained from the Sandwell gravity model.

In this paper, the Sandwell global gravity model was used for gravity compensation. This model complies with the new satellite altimeter from Gryosat-2 and Jason-1, which can provide precise gravity information over the oceans. Since the gravity data in the Sandwell model are stored as grids with the resolution 1 arcmin [32], a simple bilinear interpolation method was chosen to obtain the DOV value not located on the grids. The DOV along the track is shown in Figure 10, where the peak value could reach 40 arcsecs and changed sharply in 10–25 h and relatively gently in other segments.

**Figure 11:**
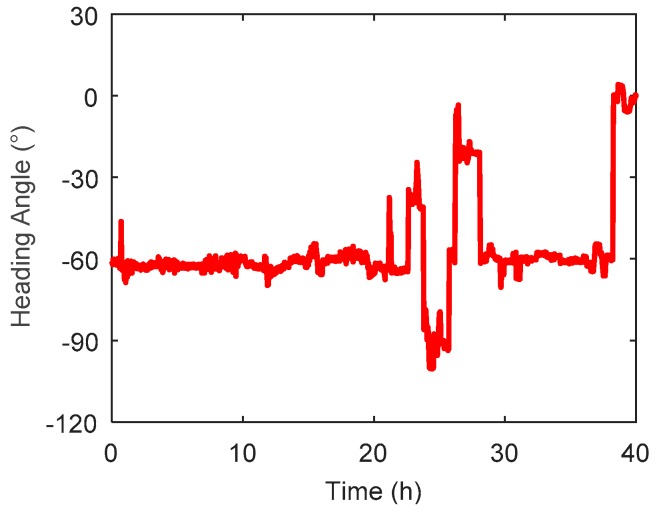
The heading angle of survey ship along the survey line.

**Figure 12:**
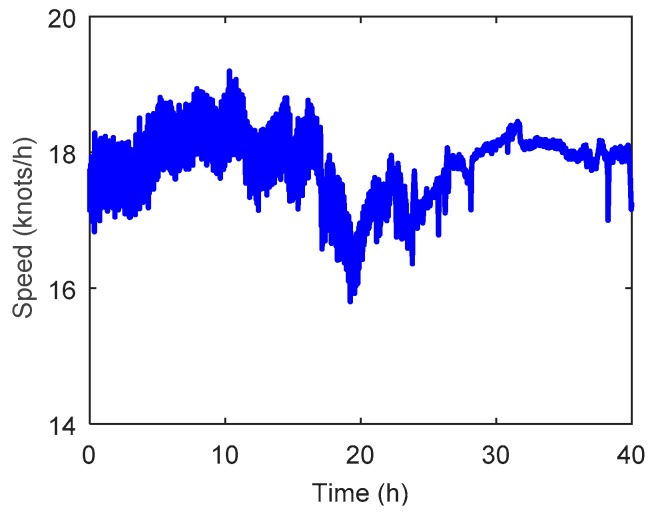
The travel speed of survey ship along the survey line.

The heading angle of the survey ship is shown in Figure 11, where it can be seen that the survey ship had several heading motions, especially in 20–30 h, and the change could reach 80°. Figure 12 shows the travel speed of the survey ship, where the average speed was about 18 knots/h (9 m/s) and had no significant variations during the whole marine test.

In the marine test, a single-axis rotation INS was mounted on the survey ship and integrated with GNSS. The specifications of the IMU are listed in Table 2. 

For the GNSS, a NavCom SF-3050 receiver with a multi-constellation antenna was mounted on the upper deck of the survey ship. Due to the lack of local base stations in the remote sea, the StarFire^TM^ global subscription service was purchased to provide a real-time precise point positioning (PPP) service [33], which can achieve decimeter level positioning accuracy. The specifications of the GNSS receiver are listed in Table 3.

**Figure 13:**
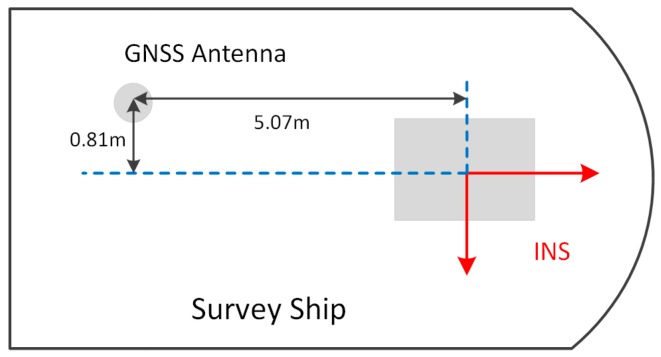
The configuration of the GNSS antenna and single-axis rotation INS in the marine test.

The configuration of the INS and GNSS is shown in Figure 13, where the lever arm between the GNSS antenna and INS was −0.81 m, −5.07 m, and 7.26 m in the ship body frame, which had been previously measured by an electric total station. The velocity and position errors caused by the lever arm were corrected in the INS/GNSS integration algorithm.

### 4.2. Data Processing

**Figure 14:**
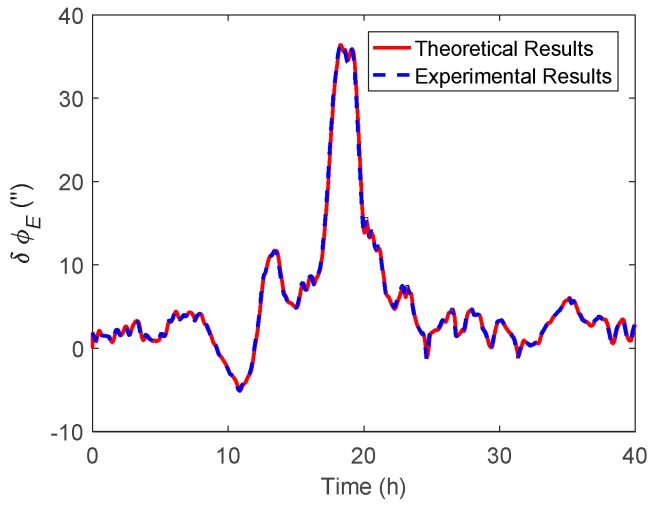
The east component of the attitude difference between the results with and without DOV compensation.

**Figure 15:**
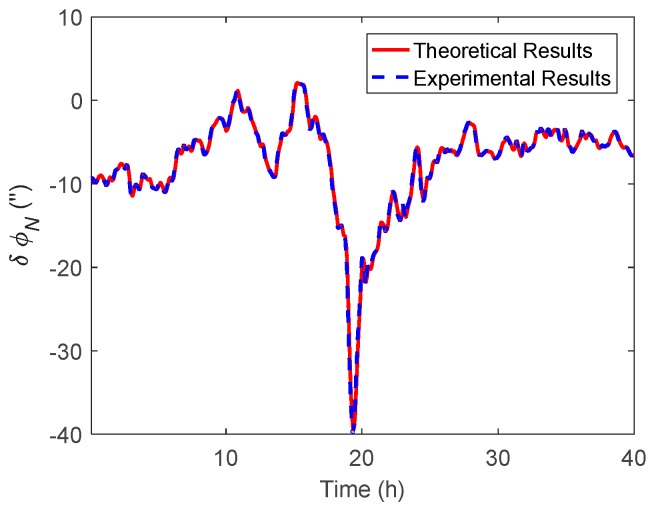
The north component of the attitude difference between the results with and without DOV compensation.

**Figure 16:**
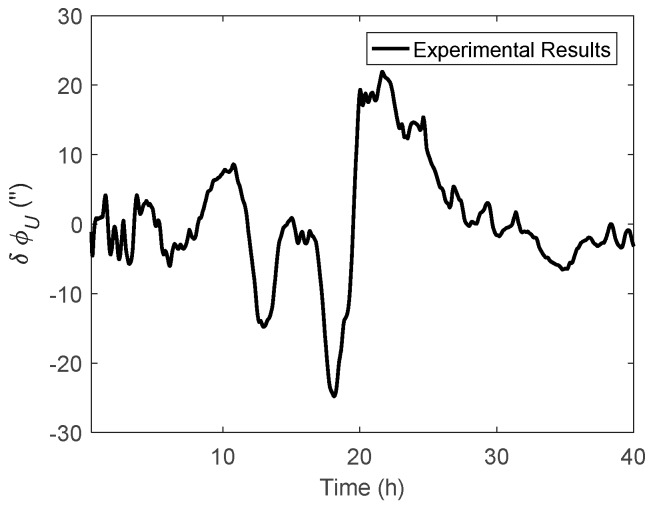
The heading difference between the results with and without DOV compensation.

In the marine test, the raw inertial and GNSS data were collected for post-processing. The attitude results with DOV compensation were compared with the corresponding results where compensation was not used, so that the effect of DOV on the attitude estimation could be evaluated. As mentioned in the above subsection, as the gravity data in the Sandwell model are stored as grids, the DOV along the track could be obtained through the use of the bilinear interpolation method. Subsequently, the normal gravity will be revised by the DOV value in the velocity update of the INS, after that, the DOV compensation can be accomplished. The east and north components of attitude difference are shown in Figure 14 and Figure 15, respectively, furthermore the theoretical results were also performed in them. Figure 16 shows the heading difference. 

Like the simulation, the theoretical results represent the value calculated by Equations (11) and (12), while the experimental results were the attitude difference between the results of the navigation Kalman filter with and without DOV compensation. As shown in the experimental results of Figure 14 and Figure 15, the peak of $\delta \phi_{E}$ could reach 35 arcsecs and $\delta \phi_{N}$ could also reach −40 arcsecs. The experimental results were well consistent with the theoretical results. From Figure 16, it can be seen that the heading difference fluctuated with time and could exceed 20 arcsecs easily, which indeed confirms that the heading estimation can be affected by the DOV along the track. Thus, in high-precision application cases, the effect of DOV must be considered to achieve preferable attitude information. 

**Figure 17:**
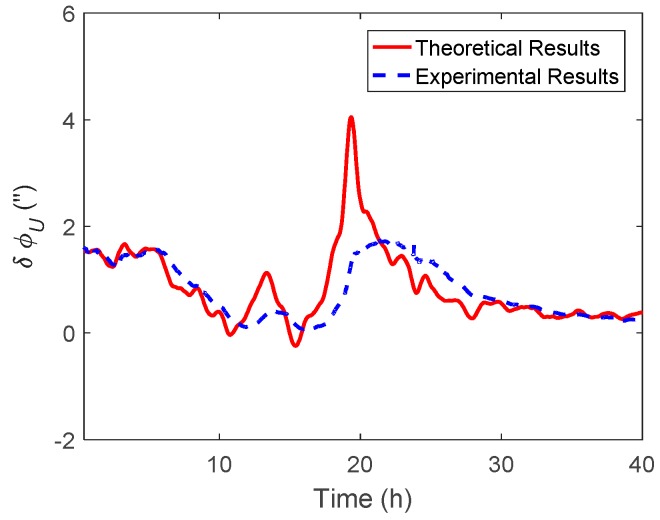
The heading difference between the results with and without η compensation.

**Figure 18:**
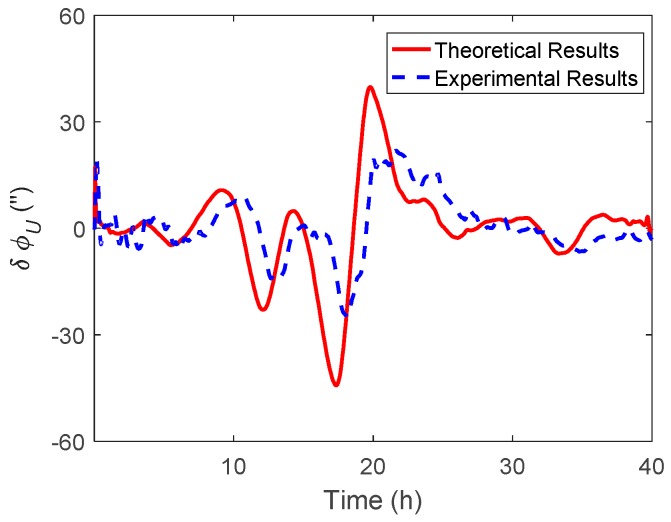
The heading difference between the results with and without ξ compensation.

Subsequently, the ξ and η compensation were implemented separately in the INS/GNSS integration, which aimed to investigate the effect of the different components of DOV to heading estimation. The corresponding theoretical and experimental results are shown in Figure 17 and Figure 18.

As shown in the experimental results of Figure 17 and Figure 18, the heading difference caused by η was less than 2 arcsecs, while that caused by ξ could exceed 20 arcsecs, which means that the heading estimation was more sensitive to ξ than η in this marine test. The theoretical results in Figure 16 and Figure 18 can be calculated by Equations (28) and (29), respectively. Since the DOV signal is rough with time, the differential operation in the theoretical calculation can enlarge the noise of the DOV. Thus, the theoretical results of the heading estimation error were previously smoothed. As shown in Figure 17 and Figure 18, although there was a slight difference between the theoretical and experimental results, their tendency with time were coincident, which was also consistent with the above simulation results.

## 5. Conclusions

This paper focused on the attitude estimation error caused by DOV in a high-precision INS/GNSS integrated navigation system. The simulation results showed that the existence of DOV along the track could not only introduce the tilt error of platforms, but that the heading estimation could also be affected due to the intercoupling characteristic between the attitude errors. Furthermore, the real shipborne marine test confirmed this concept further, where the heading estimation error caused by DOV could easily exceed 20 arcsecs. Thus, in application cases where high-precision attitude information is demanded, the effect of DOV must be considered to achieve the preferable results.

## Figures and Tables

**Table 1:** The quality of the simulated inertial sensors.

**INS**	**Error items**	**Bias**	**White noise**
	Gyros	0.003 °/h	0.0005 °/√s
	Accelerometers	10 mGal	10 mGal/√s
**GNSS**	**Error items**	**Position**	**Velocity**
	Horizontal	2 m	0.03 m/s
	Vertical	4 m	0.06 m/s

**Table 2:** The high-precision IMU specification.

	Gyro	Accelerometer
Bias instability	0.003 °/h	10 mGal
Random Walk	0.0005 °/√h	10 mGal.√s
Scale factor instability	<5 ppm	<5 ppm
Data update rate	1 kHz	

**Table 3:** The NavCom SF-3050 receiver specification.

**Position accuracy** **(one-sigma)**	Horizontal axis	5 cm
	Vertical axis	10 cm
**Velocity accuracy** **(one-sigma)**	Horizontal axis	0.03 m/s
	Vertical axis	0.06 m/s
**Data update rate**	20 Hz	
